# Topics of Public Interest

**Published:** 1918-03

**Authors:** 


### TOPICS OF PUBLIC INTEREST

---

**Mobilizing the Profession for War.** Lt. Col. Robert E. Noble, M. C., emphasizes in the Dec. issue of the Southern Medical Journal the need of the country. The Medical Reserve Corps is, at present, just about adequate to the immediate needs of the troops raised. The Surgeon General should have men ready, either in training or available for immediate special detail from civil life as units of troops are raised. A million more troops will require about 10,000 more physicians. We have already alluded to the fact, which Col. Noble states in slightly different terms, that the best preliminary examining and exemption board is the physician himself. Every young man as he completes his interneship should jump at the opportunity, unless he is physically deficient. A first lieutenancy in the army is the best experience offered and at the highest pay, unless in very exceptional instances. It is likely that the next graduating class will be required without interne service, for there is great need of the equivalent of internes in the army, to relieve older and more experienced men of details. But, probably not more than 2,500 medical officers a year can be drawn from any one graduating class, and the army needs somewhere in the neighborhood of 10,000 physicians, at least where it can put its fingers on them. So far as our observation goes, the M. R. C. is divided just about 33 1/3—33 1/3—33 1/3 among the young men, the men of 35-45 and the men well over draft age for any country. The older men seem to stand army life and to enjoy the many social pleasures afforded just as well as the younger ones. Good use can be made of any kind of specialist, even the obstetrician and gynecologist, and the government can convert a general practitioner into a specialist and vice versa, or turn one kind of a specialist into another with the greatest facility, either by actual clinical work under proper guidance, or by sending him to some medical center for a special course. Even if a physician is modest enough to think that he does not amount to much as such, there are plenty of needs for mere faithfulness, common sense, or ability along other lines, to carry on the manifold functions of the medical department. But don't volunteer if you are afraid of hard work, or if you are going to insist on being pampered or paid on the basis of fancy consultation prices, or if you expect to be continually thanked for the sacrifice you will have made.

---

**Tuberculosis of Stock Animals.** Dr. J. A. Kiernan, Chief of the Tuberculosis Division of the Bureau of Animal Industry, states that, of 14 million cattle and sheep slaughtered during the fiscal year ending with June, 1917, 3,397,000 were tuberculous. He thinks that swine are infected from skim milk returned from creameries, and that this by-product should be pasteurized. With the growing belief that so-called tuberculin reactions are merely due to sensitiveness to foreign proteins, more common in the tuberculous but not really diagnostic, the problem becomes increasingly difficult. However, human tuberculosis seems to be on the decline and it is possible that there is some fallacy in this alarm.

**The Dunkirk Health Dept.** gives the following vital statistics for 1917: births 596, deaths 309, the highest for each in 6 years. 178 marriages took place, the largest number since 1913.

---

**The U. of B. Pharmacal Alumni** held their annual dinner at the Ellicott Club Jan. 31.

---

**Jackson Health Resort** at Dansville is now a government hospital with Major Arthur H. Crosbie of Boston in charge. It will be known as hospital No. 18. Soldiers and officers of the U. S. from home and abroad will be received as patients. The outlying cottages will be used more particularly for officers, soldiers and nurses, the patients to be cared for in the sanitarium proper. When improvements are completed there will be accommodation for 500.

---

**The New City Hospital** on the West Farm site in Kensington was opened for the care of tuberculous patients. This will help to relieve the crowded condition of the J. N. Adam Hospital at Perrysburg.

---

**The Minnesota State Pharmaceutal Association** convention was held Feb. 6-8, 1918. F. A. Upsher Smith, Ph.C., of St. Paul, read a paper on the conservation of glycerin, sugar and alcohol in medicinal use. He said that the British Pharmaceutical Society has worked out changes in the formulas in the British Pharmacopia to reduce the consumption of sugar and glycerin. These modified formulas have been officially adopted by the Pharmacopia committee of the General Medical Council for the duration of the war, and this body has withdrawn from the Pharmacopia directions for the use of glycerin and sugar in certain preparations. Mr. Smith urges that the U. S. pharmacists follow the example of their British colleagues. He figures that the U. S. pharmacists use yearly 2 million pounds of glycerin and 500 pounds of sugar, and that a very large amount of this material could be saved for other purposes.

---

**Mr. R. R. Denny,** chairman of the Physician's Lease Committee of the Chicago Rotary Club, announces that Senate Bill No. 2859, providing a moratorium for soldiers and sailors during the war, has been passed. Doctors are urged to procure a copy of this bill and see how it applies to the subject of physician's leases.

---

**The Avon Inn, for many years a sanitarium, noted for its**

sulphur baths, more recently an automobile inn, will shortly be opened as a sanitarium by Dr. W. E. Gregory for many years of the staff of the Jackson Health Resort of Dansville, associated with Dr. Glidden, who joined the staff of the latter institution a few years ago. The Jackson Health Resort will be taken over by the U. S. Government.

---

**Veterinarians Needed for Army Medical Corps.** About 2,000 trained men are needed for foreign service.

---

**The Corning Hospital** will receive $100,000 from the estate of the late Miss Etha Bump.

---

**The State Sanitary Code** has been adopted by the town and village of Lancaster and the town of Elma.

---

**The Buffalo Base Hospital Unit** has been divided into two sections in France.

---

**The Rochester Base Hospital No. 19**, was given a farewell dinner at the Genesee Valley Club, Jan. 2, by the Rochester Medical Assn.

---

**N. Y. State to be Dry.** A bill has been introduced to make the state bone dry, except the use of liquors for medical, scientific and sacramental purposes, from Oct. 1 for the duration of the war and a year allowed for demobilization.

---

**Daylight Saving.** It is expected that the state legislature will pass a bill setting the clocks forward one hour from April to September. The fact that such a bill is necessary to conserve light (and power) and individual eye-sight and domestic expense shows what children we are. Why can we not simply get up and go to bed earlier? But, if we are going to tinker with the clocks at all or imagine ourselves 15 degrees east of where we live, why not go further and let every one enjoy the luxury of rising at 9 and retiring with the pride of being a rounder?

---

**War Course in Dentistry.** The American Institute of Dental Teachers, at its recent meeting in Pittsburg, has appointed a committee to formulate a standard course for dental colleges. Dr. Abram Hoffman of Buffalo is the Secretary. One of the things that will go far toward making the present war pay for itself is the routine reconstruction of men, with reference to teeth and other organs. The direct aggregate gain in efficiency will be an enormous credit against the charge of war and the indirect example of the work done among soldiers will multiply this factor.

---

**Binoculars, Spy Glasses and Telescopes** are needed in the navy. It is hoped that these will be given to or loaned to the government at a nominal rate of $1 each. There is a scarcity in the market supply of such instruments and thousands are held by private persons who have no real use for them. It is hoped that the people will respond generously. The small sacrifice is a sort of insurance against loss of ships and men by the country. One good instrument mobilized into active service may mean the destruction of a submarine and the saving of many vessels.

---

The National Hygienic Society asks us to publish a warning against the **Common Soda Water Glass.** It cites one carelessly washed glass that yielded 20,000 human epithelial cells and had an estimated germ population of 3 million. With personal recollection of the filthiness of some very popular and by no means low priced fountains, we acquiesce but we also wish to protest against the profiteering substitute of a piece of paraffine paper which magnifies the apparent volume and affords a very doubtful assurance of protection.

---

**Bills in the Interests of Officers and Soldiers.** The Senate Military Committee has reported favorably on a number of bills, of which the following are of special interest to medical reserve officers; though applying to all officers and soldiers: reimbursement for private property lost in the discharge of duty; continuance of pay for six months, to widow, children or other designated dependent, if death results from wounds or disease not due to misconduct. Another bill provides for paying line and department officers the same as staff officers. In regard to reimbursement for loss of private property, one should remember not only the obvious risks of service at the front but that from fire in the tents and barracks employed in this country. Private insurance for short terms, liable to termination or transfer by sudden tranfer, even against fire alone, would be at prohibitive rates.

---

**Reduction of Milk Supply Due to War.** Beginning Dec. 8, the use of milk and cream in Great Britain is limited to infants, invalids and butter makers. The retail price of milk has, however, advanced only to 14-16 cents a quart. (Imperial or American not stated; if the former, the increase is only to about 12-14 cents). All over Europe, the reduction in milk production has been relatively greater than that of cattle, owing to forage conditions and the increased shortage of meat. It is said that in France, the reduction in number of cattle is 1/7, of Milk, to 40% of the normal and that in Vienna, the milk supply has diminished from 900,000 to 200,000 liters. In Berlin, the milk supply is said to be almost exactly 1/3 of the ante-bellum standard.

It is interesting to note in comparison that the high price of milk has diminished the consumption in Buffalo by about 3,000 gallons a day and that the number of dealers has been reduced from 214 to 190 in the last two months of 1917. It is worth while emphasizing the fact that milk is not an especially good food for healthy adults, and that it is now a relatively expensive food. It is the patriotic duty of husky grown men to quit using milk, plain or elaborated into various soft drinks, as a beverage. 1/2 to 2/3 of a pint a day is an adequate allowance for adults, in cooking and for use in the ordinary table beverages.

---

**The Glen Springs Sanitarium** was reported in our last issue as having been taken over by the government. Mr. Leffingwell, the President writes us that there has been no negotiation even for the leasing of the Glen Springs. The Jackson Health Resort at Dansville, which was under the same management, has been taken over by the government.

---

**Waste of Meat in War.** Since the beginning of the war, it is estimated that about 33 million meat animals have been slaughtered and the loss not made good, on account of shortage of fodder. It is further estimated that in this country, the meat ration must be reduced about 1 ounce per capita per diem, to compensate.

---

**The Third Liberty Loan** will be offered about Mch. 1. It is estimated that about 15 million individual investors will be needed. The bonds are as nearly absolutely safe as is humanly possible. They are actually better revenue producers than any other class of conservative investments, even those involving a much higher risk for the two classes of the population who have very small and very large amounts of money to invest at any given time, or are at least equal to the best rate of interest paid by sound savings banks. By combining different forms of taxation and taxation and credit, the burden of the war is distributed as generally and as equably as possible throughout the population and over a period of years. By allowing payment to be made in installments, personal and banking monetary stringency is prevented and the money raised is returned in various channels so as to keep it in circulation, with a minimum reserve. The taxes collected and the loans secured by the government constitute a sort of portal circulation to nourish the vital organs of the government and the precautions just mentioned prevent the possibility of bleeding the country to death into its own portal circulation. In addition, the rapid return from this portal to the general monetary circulation enriches the latter and it is by no means impossible that in spite of the obvious losses of war, a wide spread condition of heightened prosperity may be continued. We should firmly grasp the idea that money by itself is worthless. This country has more money of all kinds, actually and per capita than any other. What it needs is labor and material. Hence it is by no means undesirable that much individual sacrifice should be involved in subscriptions to the Liberty Loan. Such sacrifice means that labor at present devoted to catering to unnecessary luxury and amusement should be forced into immediately needed production and that clothing, food and various other materials beyond the actual needs of the individual should be available for the men actually engaged in the war. Even if the majority of the materials are not directly available for combatants, they are indirectly available in the raw or partially prepared state.

---

**The Peanut Crop of Alabama, 1917,** is estimated at 16 million bushels or 200,000 tons. This would furnish about 300,-000,000 day's rations of 2500 calories each and would support the entire population of the country for 3 days.

---

**The Niagara Co. Tuberculosis Hospital** will be opened this spring in Lockport. The cost was $100,000. Among the managers are Drs. Harry C. Dumville of Niagara Falls and C. T. Crance of N. Tonawanda.

---

**Superiority of Country Boys a Myth.** Contrary to the expectation at the time, it was found early in the Civil War, that boys from cities made better soldiers than country boys, largely on account of psychic factors. Many hold that the same holds good today. At any rate, on a purely physical basis, 35,017 city registrants gave 28.47% of rejections while 44,462 from rural districts gave 27.96%, virtually a tie.

---

**Buffalo Vital Statistics, 1917.** Births, 13,536, an increase of 463 over 1916; deaths 7,537, an increase of 58.

---

**Automobile Statistics,** 1916 and 1917. Owners, 213,548 and 407,022. Dealers, 2,492 and 2,727; Chauffeurs, 103,774 and

131,788. Receipts from licenses, $2,575,111.50 and $4,266,-949.50. In 1900, there were only 954 automobiles in the state. It should be noted, however, that the receipts would pay for only about 250 miles of hard surfaced roads.

---

**Football Casualties** for 1917 totaled 12, as against 18 for 1916, 16 for 1915 and 15 for 1914. None of the past year's mortality occurred in university and college teams but was confined to high schools and miscellaneous elevens.

---

**Automobile Fatalities,** 1917. 837 as compared with 729 in 1916 and 455 in 1912. The complaint of the National Highways Protective Society that no legislation of importance had been enacted, seems to us to show a misconception. Very few evils can be corrected by law alone. More general legislation, based on common sense and experience, rather than on prejudice, has already been placed on the statute books of many states and local inforcement of existing laws is both more efficient and more rational. Two very important laws have been passed, notwithstanding the statement made. The head-light law, though still subject to variation and imperfect is certainly important. It will, when worked out by experience and properly inforced, prevent numerous ditching accidents, quite a number of which have proved fatal. It will also materially reduce the deaths of pedestrians who happen to be on the road when two autos pass at night. Legislation allowing autos to pass standing street cars, at a reasonable speed, when adequate space is afforded, though, in a sense, a letting down of the bars, has lessened congestion of traffic to a considerable degree and congestion of streets is an inevitable cause of a certain number of deaths. There has also been a marked tendency to get away from the narrow conception of preventing accidents by legislation only against automobilists and a beginning has been made in the proper direction of placing due responsibility upon pedestrians and slow-moving vehicles.

---

**The Trial Trip of Sending Motor Trucks Under Their Own Power From Detroit to the Seaboard** has been successful. We venture to state that if this state had followed the policy advocated by this journal more than three years ago of concentrating its road improvement on permanent hard-surface construction and on trunk routes and if other states had followed the same policy in co-operation, this movement would have been in no sense a trial but a foregone conclusion and would have been more successful in the sense of better time and lessened strain and danger.

**The Pure Food and Drug Law.** The Bureau of Agriculture reports that over 40,000 citations of manufacturers have been made, that 750,000 shipments of domestic or imported foods and drugs have been made, that factory inspections run up into many thousands and calls attention to the vast improvement after ten years of operation of the law. Unlabeled, secret proprietary mixtures have become a matter of past history, as have the worst examples of decomposed food stuffs. The states have quite generally supplemented the Nation in passing and enforcing laws. Adulteration has been reduced to comparatively harmless practices or to those in which the greatest ingenuity has been called into play to evade the laws. Honest manufacturers and even those who are merely playing safe have in numerous instances asked the assistance of the authorities against unfair competition by those who were not complying with the laws. We can remember being called visionary for suggesting that the A. M. A. or some similar body could carry out one part of all this, by at least enabling physicians to know what patent medicines contained.

---

**Military Census of N. Y. State.** Persons between the ages of 16 and 50 number 5,815,969, approximately half of the total. Males 2,914,909; females 2,901,060, the difference being much less than has usually been believed, in view of the increase of males by immigration. In N. Y. City, females predominated, 1,650,775 to 1,630,095 males, total 3,280,870. The statistics for Western N. Y. are as follows:

| Counties. | Males. | Females. |
|---|---|---|
| Allegany | 9,288 | 9,288 |
| Cattaraugus | 20,066 | 16,740 |
| Chautauqua | 29,362 | 29,623 |
| Chemung | 15,924 | 17,276 |
| Erie | 180,351 | 170,342 |
| Genesee | 9,716 | 9,052 |
| Livingston | 9,112 | 8,492 |
| Monroe | 97,406 | 94,890 |
| Niagara | 37,863 | 29,355 |
| Orleans | 7,797 | 6,816 |
| Steuben | 21,095 | 20,481 |
| Wyoming | 7,696 | 7,718 |

---

**Horse Chestnuts** have been used in Germany for food, though the extraction of the bitter principle is not economic except under war conditions. In England, they have been successfully used as a basis of explosives, though twice the weight of grain is required. It is estimated that 25,000 tons have been gathered this year and that 8 times as much were available. Here is a practical hint to the patriotic small boys of America, for next fall.

---

**Suit for Collection of Fee for Christian Science Treatment.** What is said to be the first suit of this nature has been instituted by Miss Grace M. Trankla of N. Y., against Clarence C. Burger, for $12,150, for services and instruction between Apr. 1 and Oct. 1, 1917. This will bring up the question of the status of Christian Science from a new standpoint.

---

**Attrition Statistics.** We have in the past published various statistics of killed prisoners, wounded and incapacitated, etc., for various countries. The fallacy of these statistics has been shown by the fact that there has been not only a decided proportional decrease as the war has progressed but in some instances an actual decrease as the period of time has increased At about the conclusion of the third year of the war, Germany admitted a loss of about half a million in prisoners, altogether, though the statements of the allied countries of German prisoners held by them, came to a much larger number, 1-2 million. Recently, it has been stated that the failure of the Russian offensive would liberate 2 million German prisoners (sic, not combatants on the eastern front). A request to the Official Bulletin for accurate, up-to-date statistics, has been referred in turn to the Secretary of War and to the Librarian of Congress and the latter is unable to do more than give references, mainly of the dates of 1915 and 1916. We make this statement to show the impossibility of giving information In general, we have found that, since our entry into the war, we are getting propaganda rather than news.

---

**Advisory Boards to Pass Upon Drafted Men.** It is expected that the classification of drafted men according to dependents, etc., will be completed by Feb. 15. Adequate local boards will then be provided to make examinations for exemption of Class 1 men (and ultimately of other classes if required) with ample time for proper consideration.

---

**Socialization of the Medical Profession** of Great Britain by legislation, providing for universal free medical attendance is expected at an early date. The American profession should recognize the possibility of similar action in this country. Indeed, Health Insurance legislation in many states is either in operation or threatened and it is estimated that this will apply to 50-80% of the population. If necessitated as a war measure, it would be an unwise and a futile policy to attempt organized professional obstruction. Rather, there should be a concerted attempt to secure fair play and reasonable demands, somewhat according to the standards of the army and navy medical services.

